# Determinants of retinal and choriocapillaris microvascular alterations in pediatric β-thalassemia: an OCTA study

**DOI:** 10.1186/s40942-026-00852-z

**Published:** 2026-06-02

**Authors:** Mohamed Rateb Amin, Maha Mohamed Youssef, Amina Abdelsalam Ali, Lameece Moustafa Hassan

**Affiliations:** 1 Memorial Institute of Ophthalmology, Cairo, Egypt; 2 Faculty of Medicine, Kasr Al Ainy Hospital, Cairo University, Cairo, Egypt; 3 Ophthalmology Department, Kasr Al Ainy Hospital, Cairo, 11562 Egypt

**Keywords:** Thalassemia, OCTA, Vascular density, Hemoglobin, Ferritin

## Abstract

**Purpose:**

To evaluate retinal and choriocapillaris microvascular changes observed using optical coherence tomography angiography (OCTA) in pediatric patients with β-thalassemia and to identify hematologic and clinical predictors of these changes.

**Methods:**

This cross-sectional observational study included 120 eyes of 120 children (< 18 years): 40 with thalassemia major, 40 with thalassemia intermedia, and 40 age-matched controls. All participants received a thorough ophthalmic examination and OCTA imaging (6 × 6 mm scans) to assess vessel density (VD) in the superficial capillary plexus (SCP), deep capillary plexus (DCP), and choriocapillaris (CC), as well as foveal avascular zone (FAZ) parameters. Clinical data included hemoglobin level, serum ferritin, transfusion history, hydroxyurea use, and splenomegaly. Correlation and regression analyses were conducted to determine predictive factors of the associated OCTA changes.

**Results:**

In both thalassemia major and intermedia, the area and perimeter of the FAZ was significantly greater as compared with controls (*p* < 0.01). SCP whole VD was significantly lower in thalassemia intermedia compared with controls (*p* = 0.023), while DCP whole VD was significantly lower in both thalassemia groups (*p* < 0.001). Choriocapillaris flow was also significantly reduced in thalassemia patients (*p* < 0.001). Serum ferritin levels and blood transfusion rate showed limited and inconsistent correlations with OCTA parameters. In contrast, hemoglobin level demonstrated significant positive correlations with VD across multiple SCP and DCP regions. Multivariable regression analysis indicated that hemoglobin was the only independent predictor of superficial and deep whole VD (*p* = 0.015 and *p* = 0.002, respectively).

**Conclusions:**

Children with β-thalassemia exhibit significant subclinical retinal and choriocapillaris microvascular alterations detectable by OCTA. Hemoglobin level, rather than iron indices, is the strongest determinant of retinal vascular density, suggesting that chronic anemia-related hypoxia plays a central role in retinal microvascular compromise in pediatric β-thalassemia.

## Key messages

Both thalassemia major and thalassemia intermedia show reduced retinal vessel density and enlarged FAZ, with more pronounced involvement of the deep capillary plexus and choriocapillaris.

Hemoglobin level is the strongest independent predictor of retinal microvascular density, outperforming serum ferritin, transfusion status, and other clinical variables in multivariable analysis.

Iron overload indices show limited and inconsistent associations with OCTA parameters, suggesting that anemia-related hypoxia rather than iron burden is the primary driver of microvascular compromise in this population.

## Introduction

Thalassemia is an autosomal recessive hemoglobinopathy that arises from mutations or deletions in globin genes, leading to reduced or absent synthesis of hemoglobin globin chains. The underproduction of the alpha or beta globin chains results in alpha-thalassemia or beta-thalassemia (β-thalassemia), respectively [1–3]. β-thalassemia poses a significant public health challenge in Egypt; El-Hashemite et al., reported that from 1.5 million annual births, approximately 1,000 children have β-thalassemia major [4].

The phenotypic classification of β-thalassemia includes three forms: minor, intermedia, and major. β-thalassemia minor represents the heterozygous state and is associated with an anemia milder in nature, that not requiring blood transfusion therapy. In contrast, patients with β-thalassemia major typically develop severe anemia that necessitates repeated transfusions, which often lead to iron overload and require chelation therapy. β-thalassemia intermedia, caused by compound heterozygosity, presents with anemia of moderate severity, intermediate to the minor and major forms and typically does not require lifelong regular blood transfusions [5, 6].

Previous studies have demonstrated that several factors—including iron overload leading to vascular obstruction, chelation therapy following repeated blood transfusions, tissue hypoxia secondary to chronic anemia, and a hypercoagulable state in β-thalassemia lead to the development of ocular complications. These complications include retinal pigment epithelium (RPE) changes, optic nerve affection and alterations within the vascularity of both the retina and choroid [5, 7].

Given the potential for subclinical microvascular impairment to precede overt visual dysfunction, early detection of these changes is clinically important. Optical coherence tomography angiography (OCTA) enables noninvasive assessment the retinal and choroidal microvasculature. By offering quantitative measurements, OCTA can improve insight on the ocular pathology that may develop in patients with β-thalassemia [1, 5, 8, 9].

Despite the well-established documentation of ocular involvement in β-thalassemia [5, 7], the extent and pattern of chorioretinal microvascular alterations remain incompletely understood, particularly in relation to disease severity, transfusion status, and iron overload. Previous studies have primarily focused on structural retinal changes, while quantitative assessment of retinal and choroidal microvasculature and the relative contribution of chronic anemia, iron toxicity, and hypercoagulability to these vascular changes remain variable and not clearly defined [10, 11].

Therefore, this study aims to evaluate chorioretinal microvascular changes in patients with both β-thalassemia major and intermedia, using OCTA, and to investigate their relationship with clinical and hematological parameters.

## Methodology

This study is a cross-sectional observational study, including 120 eyes of 120 children (less than 18 years of age), 40 thalassemia major patients, 40 thalassemia intermedia and 40 age- matched controls. Thalassemia patients were recruited from the pediatric hematology department, Abou El Reesh Hospital, Cairo University. This study adheres to the tenants of the Declaration of Helsinki. A written informed consent of participation was obtained from all included participants’ guardians. The Cairo University ethics committee provided ethical clearance, (IRB approval no. MD-406-2022).

Eligible thalassemia patients all had a definite diagnosis of β-thalassemia using clinical, hematological, and electrophoretic studies (qualitative and quantitative hemoglobin analysis).

Any patient with any ocular pathology (glaucoma, uveitis, hereditary retinal disorders, etc.), previous ocular surgery or trauma, media opacity or high myopia hindering OCTA acquisition were not included. In addition, patients on deferoxamine were excluded, due to retinal toxicity and only patients on deferasirox or deferiperone were included.

Collected data included demographic characteristics, clinical characteristics and multisystem involvement of β-thalassemia (splenomegaly, stunted growth and others). Blood transfusion history (onset and frequency), history of concomitant medical conditions and drug history, including hydroxyurea, chelation therapy and others. Blood levels of hemoglobin (g/dL) and serum ferritin (ng/mL) levels were documented.

A complete ophthalmic examination and OCTA imaging (Optovue Angiovue System, software ReVue XR version 2017.1.0.151, Optovue Inc., Fremont, CA, USA) to assess the retinal vascular network was conducted for each participant.

To minimize the confounding effects of transfusion-related hematologic fluctuations, all patients (who received regular blood transfusions) were examined immediately prior to their scheduled transfusion. Blood transfusion in β-thalassemia results in a transient increase in hemoglobin levels and improvement in tissue oxygenation and generally persist over the inter-transfusion interval, which is typically 2–4 weeks in transfusion-dependent patients [12].

Macular vessel density (VD) was evaluated using the AngioAnalytic™ software integrated with OCTA in the 6 × 6 mm scan mode. Measurements were obtained for the superficial capillary plexus, deep capillary plexus, and choriocapillaris. Vessel density was measured in the foveal, parafoveal, and perifoveal regions, while the software automatically subdivided the parafoveal and perifoveal areas automatically divided into superior, inferior, nasal and temporal quadrants. VD was defined as the percentage of the scan area containing blood vessels. The foveal avascular zone (FAZ) area and perimeter were automatically calculated in square millimeters the SCP. All scans were processed using the three-dimensional projection artifact removal (3D PAR) algorithm to enhance image quality and reduce projection artifacts.

### Statistical methods

SPSS software (version 28) was utilized. Data distribution and normality were calculated by the Kolmogorov–Smirnov test. Mann- Whitney U test assessed differences between two independent groups and Kruskal-Wallis test for more than two groups, followed by Bonferroni-adjusted post hoc analysis. Associations between quantitative variables were examined using Spearman’s correlation coefficient. independent factors affecting vessel density and choriocapillaris parameters were identified by multiple linear regression analysis. All tests were two sided with a p-value ≤ 0.05 considered statistically significant.

## Results

The study included 120 children: 40 patients with thalassemia intermedia, 40 with thalassemia major, and 40 age- matched controls. There were no statistically significant differences in age or sex (Table 1).

The range of best-corrected visual acuity was 0.3 to 0.0 logMAR, with no significant differences between the groups. Fundus examination revealed normal findings in all participants, except for three cases exhibiting myopic changes, including tessellated fundus appearance, peripapillary crescent, and peripheral retinal pigment epithelium (RPE) changes.

**Fig. 1:**
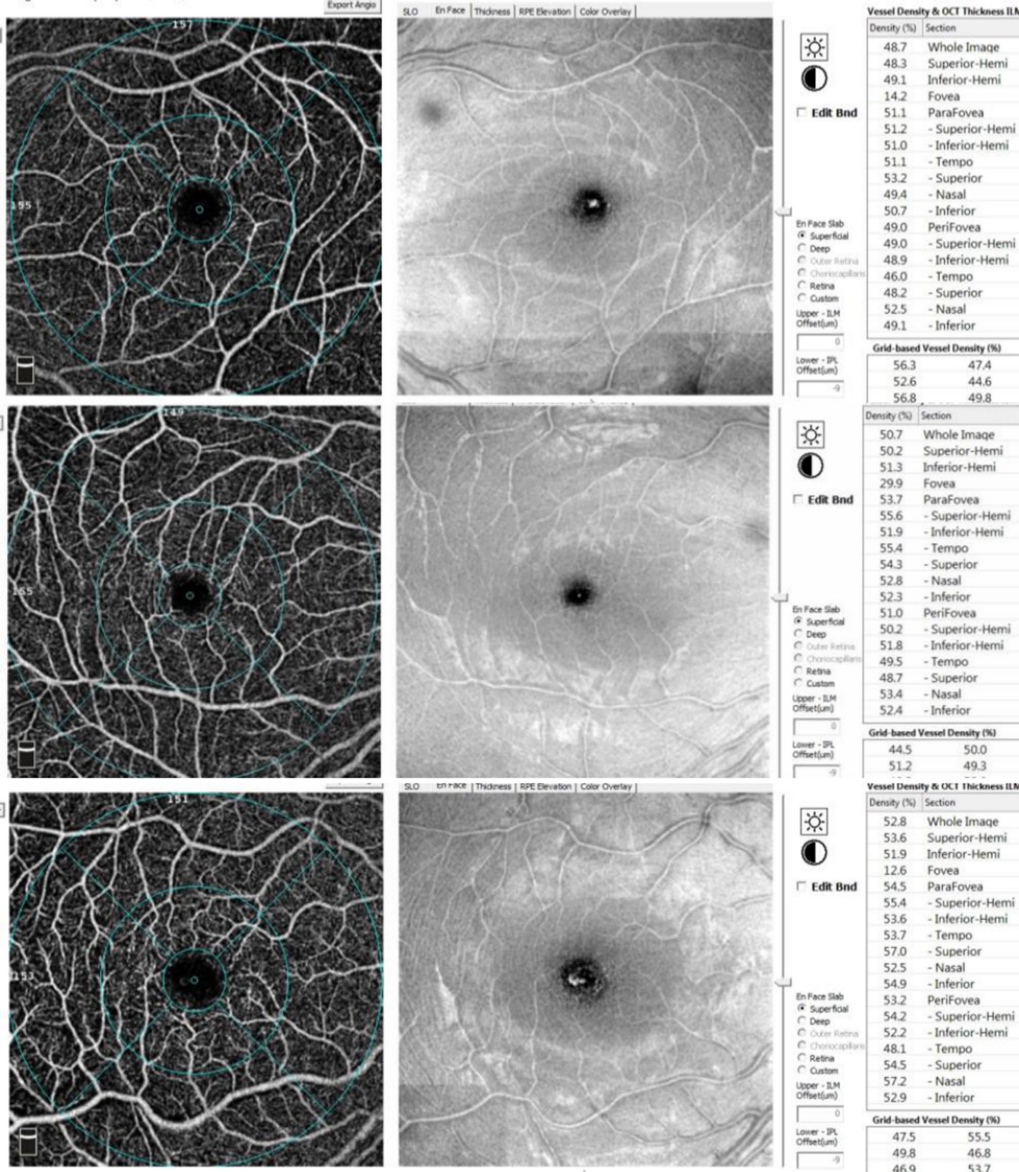
En face OCTA scans of the SCP of a case of thalassemia major (top), thalassemia intermedia (middle) and control (bottom)

**Fig. 2:**
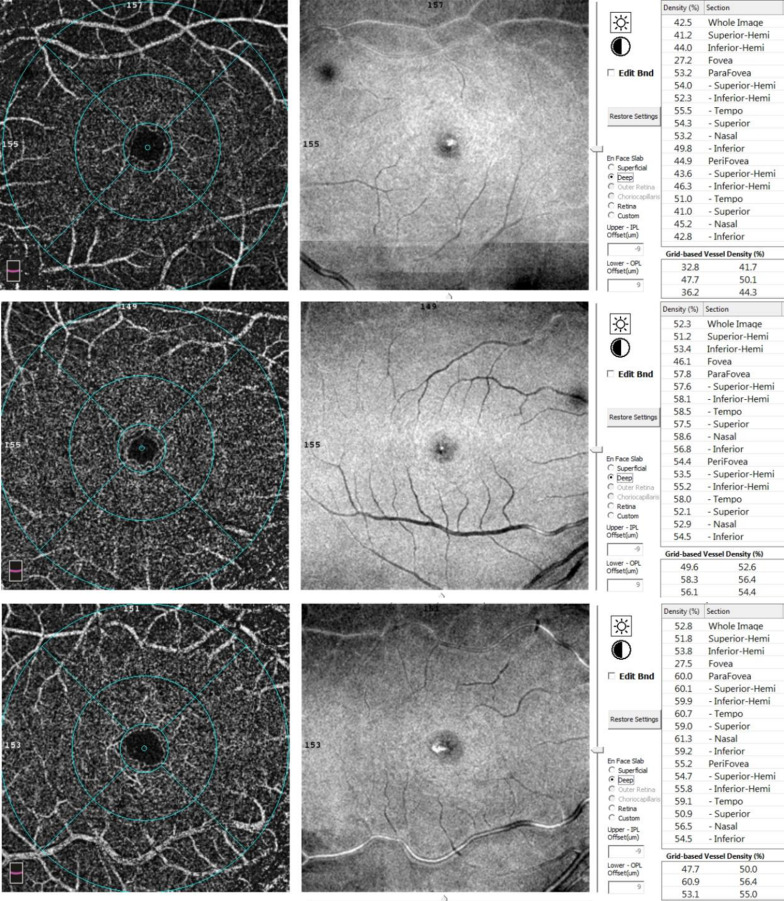
En face OCTA scans of the DCP of a same case of thalassemia major (top), thalassemia intermedia (middle) and control (bottom)

Hemoglobin levels were lower in the major group as compared with the intermedia group (*p* = 0.024). Correspondingly, patients with thalassemia major required significantly higher total transfusion volumes and more frequent transfusions, with all patients receiving monthly transfusions compared with only 15% of those with thalassemia intermedia (*p* < 0.001). Elevated serum ferritin levels were more pronounced in patients with thalassemia major (55%) (p < 0.001). Splenomegaly was observed in 47.5% of patients with thalassemia major, whereas none of the thalassemia intermedia patients exhibited splenomegaly (*p* < 0.001) (Table 1) (Figs. 1 and 2).

The parameters of the FAZ varied significantly among the three groups. Both FAZ perimeter and FAZ area were significantly smaller in the control group compared with the thalassemia major and thalassemia intermedia groups (adjusted *p* < 0.001 and *p* = 0.005, respectively, and adjusted *p* < 0.001 and *p* = 0.006, respectively) (Table 2).

Within the SCP, the whole-vessel density varied significantly among the groups (overall *p* = 0.025). Pairwise comparisons demonstrated that the thalassemia intermedia group had significantly lower whole, superior and inferior parafoveal vessel density compared with the control group (adjusted *p* = 0.023, *p* = 0.010 and *p* = 0.003, respectively). Statistical significance was not seen in the other regions (Table 2).

**Table 1:** Baseline demographic and clinical characteristics of the study groups

Variable	Thalassemia Intermedia (*n* = 40)	Thalassemia Major (*n* = 40)	Control (*n* = 40)	*P*-value
**Age (years)**,** mean ± SD**	9.2 ± 3.2	11.3 ± 3.6	10.8 ± 2.7	0.052
≤ 10 years, n (%)	25 (62.5%)	16 (40%)	20 (50%)	0.131
> 10 years, n (%)	15 (37.5%)	24 (60%)	20 (50%)	
**Gender**,** n (%)**				0.365
Female	19 (47.5%)	18 (45%)	24 (60%)	
Male	21 (52.5%)	22 (55%)	16 (40%)	
**Hemoglobin (g/dL)**,** mean ± SD**	7.8 ± 1.4	7.3 ± 1.0	—	0.024
**Blood transfusion frequency**,** n (%)**			—	< 0.001
Monthly	6 (15%)	40 (100%)	—	
Every 3 months	21 (52.5%)	0 (0%)	—	
Occasionally	13 (32.5%)	0 (0%)	—	
**Serum ferritin (ng/mL)**,** mean ± SD**	857.4 ± 826.1	2523.1 ± 2065.1	—	< 0.001
Normal, n (%)	15 (37.5%)	0 (0%)	—	
Mild to moderate, n (%)	22 (55%)	18 (45%)	—	
Severe, n (%)	3 (7.5%)	22 (55%)	—	
**Splenomegaly**,** n (%)**	0 (0%)	19 (47.5%)	—	< 0.001

As compared to controls, the whole DCP VD in the thalassemia groups was significantly lower (adjusted *p* < 0.001). Furthermore, the DCP vessel density in the nasal and inferior quadrants was also significantly reduced in both patient groups as compared to controls (adjusted *p* < 0.001 and *p* = 0.008, and *p* < 0.001 and *p* = 0.002, respectively) (Table 2). 

**Table 2:** OCTA parameters in study groups

Parameter	Thalassemia Intermedia (*n* = 40)	Thalassemia Major (*n* = 40)	Control (*n* = 40)	*P*-value
FAZ Area	0.20 ± 0.10	0.30 ± 0.10	0.17 ± 0.07	**< 0.001**
FAZ Perimeter	1.80 ± 0.30	2.10 ± 0.50	1.60 ± 0.30	**< 0.001**
**Superficial Capillary Plexus (VD)**				
Whole	48.9 ± 3.0	49.5 ± 3.2	51.4 ± 4.3	0.025
Temporal	52.2 ± 4.1	51.9 ± 5.0	51.5 ± 3.4	0.794
Superior	49.9 ± 5.0	52.3 ± 4.8	52.8 ± 4.1	0.009
Nasal	51.2 ± 4.5	51.5 ± 5.4	51.1 ± 5.4	0.984
Inferior	49.0 ± 4.2	51.3 ± 4.5	52.3 ± 5.5	0.003
**Deep Capillary Plexus (VD)**				
Whole	47.2 ± 3.6	45.2 ± 4.5	54.9 ± 2.6	**< 0.001**
Temporal	56.3 ± 4.3	54.2 ± 3.6	56.1 ± 4.7	0.052
Nasal	54.6 ± 4.4	53.4 ± 2.7	57.2 ± 4.6	**< 0.001**
Inferior	51.5 ± 4.6	51.8 ± 3.6	55.5 ± 3.2	**< 0.001**
Superior	51.5 ± 5.2	49.7 ± 6.1	55.5 ± 4.9	**< 0.001**
**Choriocapillaris Flow Rate**	2.0 ± 0.10	1.9 ± 0.20	2.2 ± 0.08	**< 0.001**

Choriocapillaris flow rate was significantly lower in both thalassemia major and thalassemia intermedia groups compared with the control group (adjusted *p* < 0.001).

Patients with thalassemia (major and intermedia) were further stratified according to serum ferritin levels into three groups: normal (≤ 300 ng/mL), mild to moderate elevation (300–2000 ng/mL), and marked elevation (> 2000 ng/mL). Analysis of OCTA parameters demonstrated no statistically significant differences. However, the normal ferritin group showed a slightly higher mean choriocapillaris flow rate compared with the other groups, approaching borderline statistical significance (*p* = 0.052).

Additionally, thalassemia participants were stratified into transfusion-dependent (monthly transfusions) and non–transfusion-dependent groups. The transfusion-dependent group (*n* = 46) included all patients with thalassemia major and six patients with thalassemia intermedia. Among the evaluated OCTA parameters, the only significant difference was a reduced vessel density in the superior and inferior SCP regions in the non–transfusion-dependent group (*p* = 0.049 and *p* = 0.005, respectively).

Correlation analyses demonstrated distinct relationships between iron indices and retinal microvascular parameters. Ferritin showed limited and largely non-significant associations with OCTA metrics with significant correlations only in the nasal quadrant of the SCP (*r* = 0.272, *p* = 0.014) and temporal quadrant of the DCP (*r* = − 0.223, *p* = 0.047), indicating weak to moderate associations.

In contrast, hemoglobin demonstrated consistent and statistically significant positive correlations with multiple OCTA measures. Hemoglobin showed significant positive correlations in the SCP across several regions, including the whole image (*r* = 0.335, *p* = 0.003), temporal (*r* = 0.435, *p* < 0.001), superior (*r* = 0.380, *p* = 0.001), and nasal regions (*r* = 0.317, *p* = 0.005). Likewise, in the DCP there were significant correlations in the whole image (*r* = 0.381, *p* = 0.001), temporal (*r* = 0.454, *p* < 0.001), inferior (*r* = 0.396, *p* < 0.001), nasal (*r* = 0.344, *p* = 0.002), and superior regions (*r* = 0.427, *p* < 0.001).

Multivariable regression analyses assessing age, hemoglobin level, ferritin level, hydroxyurea use, splenomegaly, and transfusion frequency revealed that hemoglobin level was the only independent predictor of superficial and deep whole vascular density, with significant positive associations (superficial: β = 0.285, 95% CI: 0.147–1.308, *p* = 0.015; deep: β = 0.363, 95% CI: 0.468–2.057, *p* = 0.002). There were no significant associations between the other variables and superficial or deep vascular density (all *p* > 0.05). For deep, whole and choriocapillaris vascular density, none of the assessed variables demonstrated a statistically significant association (*p* > 0.05) (Table 3).

**Table 3:** Multivariable regression analysis of factors associated with retinal and choriocapillaris vascular density

Predictor	Superficial VD (Whole Image) β (95% CI)	*p*-value	Deep VD (Whole Image) β (95% CI)	*p*-value	Choriocapillaris Flow Rate β (95% CI)	*p*-value
Age (years)	−0.164 (− 0.351 − 0.061)	0.166	0.175 (− 0.072–0.492)	0.141	0.194 (− 0.002–0.017)	0.126
Splenomegaly	0.099 (− 0.958–2.391)	0.396	0.015 (− 2.140–2.441)	0.896	−0.055 (− 0.094 − 0.060)	0.659
Hydroxyurea use	−0.122 (− 3.318–1.814)	0.561	0.030 (− 3.261–3.761)	0.887	0.041 (− 0.107–0.129)	0.854
Blood transfusion rate	−0.239 (− 4.029–1.069)	0.251	0.135 (− 2.347–4.627)	0.516	0.102 (− 0.090–0.144)	0.646
Hemoglobin (g/dL)	0.285 (0.147–1.308)	**0.015**	0.363 (0.468–2.057)	**0.002**	0.137 (− 0.012–0.042)	0.267
Ferritin	−0.118 (− 0.001–0.001)	0.410	0.024 (− 0.001–0.001)	0.868	0.024 (0.000–0.000)	0.874

β = standardized regression coefficient; CI = confidence interval

## Discussion

β-thalassemia is highly prevalent across the “thalassemia belt,” extending from the Mediterranean region traversing the Middle East to South/Southeast Asia. Within this region, Egypt represents one of the countries with the highest disease burden, with reported β-thalassemia carrier rates ranging from 9% to 10%. This high prevalence reflects the country’s geographic position and historical genetic admixture and underscores the importance of investigating systemic and ocular complications of the disease in Egyptian pediatric populations [4, 13]. 

The pathophysiology of β-thalassemia is defined by ineffective erythropoiesis, chronic anemia, and progressive iron overload. Excess unpaired globin chains induce oxidative damage to erythrocyte membranes, leading to hemolysis and further aggravation of anemia. Compensatory increases in iron absorption and frequent blood transfusions contribute to iron accumulation in multiple organs, promoting free radical generation and oxidative stress. Thus, the combined effects of chronic anemia, iron-induced oxidative injury, and tissue hypoxia seen in thalassemia major leads to impaired microvascular integrity, providing a possible mechanism for retinal and choroidal vascular alterations [14].

In our study, we evaluated retinal and choriocapillaris microvasculature using OCTA in 80 children with β-thalassemia (40 major and 40 intermedia) compared with 40 age-matched healthy controls. As expected, There were notable differences in hematological profiles across the groups, especially in hemoglobin levels, transfusion burden, and serum ferritin concentrations.

Our results demonstrated significant enlargement of the area and perimeter of the foveal avascular zone (FAZ) in both thalassemia major and thalassemia intermedia compared with controls. Previous studies have reported inconsistent findings regarding FAZ changes in β-thalassemia. Georgalas et al. did not find significant differences in FAZ measurements between thalassemia major patients and controls, whereas Cennamo et al. reported FAZ enlargement, notably affecting the deep capillary plexus (DCP) [9, 10]. The discrepancy between studies may be related to differences in patient age, disease severity, imaging protocols, and OCTA devices. Although our OCTA system did not allow separate measurement of deep FAZ, the observed FAZ enlargement likely reflects underlying capillary dropout and ischemic damage, consistent with the concept that it represents an early marker of retinal microvascular compromise.

Analysis of vessel density revealed significant reductions in both the superficial and deep capillary plexuses in those with thalassemia as compared with healthy controls, with more pronounced involvement of the DCP. Comparable results have been described in prior OCTA studies [10, 11, 15]. The DCP is characterized by lower perfusion pressure and slower blood flow, rendering it more susceptible to ischemic injury. Additionally, impaired autoregulation and localized vascular remodeling may contribute to capillary rarefaction and reduced vessel density in deeper retinal layers [16].

Choriocapillaris flow was reduced significantly in both thalassemia major and intermedia groups as compared with controls, supporting the concept that microvascular alterations in β-thalassemia extend beyond the retinal circulation. Reduced choriocapillaris perfusion may further compromise outer retinal oxygenation and contribute to subclinical structural and functional changes [10].

When patients were subdivided according to transfusion status, significant differences were observed primarily in SCP vessel density, with lower superior and inferior SCP densities in the non–transfusion-dependent group. No significant differences were detected in DCP parameters between both groups. While some studies have demonstrated greater DCP involvement in non–transfusion-dependent patients [16], these discrepancies may reflect variations in transfusion frequency, disease severity, chelation protocols, and study definitions of transfusion dependence. In our cohort, transfusion-dependent patients received regular monthly transfusions, whereas other studies have included patients receiving less frequent transfusions, potentially accentuating microvascular differences [16].

All non–transfusion-dependent patients in our study were treated with hydroxyurea. Although fetal hemoglobin levels were not directly measured, hydroxyurea therapy may have contributed to improved retinal microvascular parameters through increased HbF production and improved oxygen-carrying capacity, as suggested by previous studies [17].

Iron overload has long been implicated in the pathogenesis of vascular and tissue damage in β-thalassemia. However, in our study, stratification by serum ferritin levels and correlation analyses revealed limited and largely non-significant associations between ferritin and OCTA parameters. Similar findings have been reported by Kazancı et al. [19]. In contrast, other studies have demonstrated inverse correlations between ferritin and vessel density, particularly at very high ferritin thresholds [5, 16]. The inconsistent association between serum ferritin and retinal microvascular changes may be due to ferritin’s limited ability to reflect true tissue iron burden, as well as the confounding effects of chelation therapy and fluctuations in iron exposure over time [18].

In contrast, multivariable regression analysis demonstrated that hemoglobin level was the only independent predictor of superficial and deep retinal vascular density, after adjustment for age, ferritin, hydroxyurea use, splenomegaly, and transfusion frequency. Elevated hemoglobin levels were linked to greater vessel density in different retinal regions. of the SCP and DCP, suggesting that chronic anemia-related hypoxia may play a more central role than iron overload in retinal microvascular alterations in pediatric β-thalassemia. Similar associations have been reported in transfusion-dependent β-thalassemia and other hemoglobinopathies [11, 16].

Our findings are in line with prior studies involving hematological conditions other than thalassemia. Jian et al., found that hemoglobin levels were positively associated with retinal blood flow density on OCTA, indicating that systemic hemoglobin may influence retinal microvascular circulation [20]. Increased hemoglobin concentration improves oxygen delivery to tissues and may therefore support better retinal perfusion, which provides a physiological explanation for our results.

In contrast, other clinical variables such as iron overload parameters or transfusion history did not show a significant association in our model. Hemoglobin level represents a more immediate and physiologically relevant determinant of tissue oxygenation and retinal perfusion, whereas ferritin reflects cumulative iron burden and may be affected by inflammatory and systemic factors, thereby limiting its direct association with microvascular changes [21].

Limitations of this study include its cross-sectional design, which precludes assessment of longitudinal microvascular changes, and recruiting patients from only one center, which can impair generalizability. Additionally, deep FAZ measurements were not separately analyzed due to device limitations. Future multicenter, longitudinal studies incorporating standardized transfusion timing, direct assessment of HbF levels, and more advanced OCTA metrics are warranted to further elucidate the progression and clinical significance of retinal microvascular changes in pediatric β-thalassemia.

## Conclusion

Optical coherence tomography angiography (OCTA) showed significant microvascular changes β-thalassemia children, particularly in those with thalassemia major. Changes such as FAZ enlargement and lower vessel density were present despite a normal fundus appearance, highlighting the usefulness of OCTA in identifying subclinical retinal involvement. Hemoglobin level showed the strongest correlation with microvascular parameters, suggesting that chronic anemia and tissue hypoxia play an important role in these changes. OCTA may be useful for monitoring these patients over time and for identifying those at higher risk of microvascular involvement.

## Data Availability

All the data used and/or analyzed during the current study are available and can be presented by the corresponding author upon a reasonable request.

## Abbreviations

OCTA: Optical Coherence Tomography Angiography
VD: Vessel Density
SCP: Superficial Capillary Plexus
DCP: Deep Capillary Plexus
CC: Choriocapillaris
FAZ: Foveal avascular zone
Hb: Hemoglobin
β: Standardized regression coefficient
CI: Confidence interval
